# Genomics Reveals Recent Rapid Speciation of Sea Snakes of the Genus *Hydrophis* (Reptilia, Squamata, Elapidae)

**DOI:** 10.1002/ece3.71627

**Published:** 2025-06-19

**Authors:** Takushi Kishida, Rina Keboushi, Takahide Sasai, Mamoru Toda

**Affiliations:** ^1^ College of Bioresource Sciences Nihon University Fujisawa Kanagawa Japan; ^2^ Wildlife Research Center Kyoto University Kyoto Japan; ^3^ Faculty of Agriculture The University of Tokyo Bunkyo Tokyo Japan; ^4^ Graduate School of Engineering The University of Tokyo Bunkyo Tokyo Japan; ^5^ Okinawa Churashima Foundation Research Institute Motobu Okinawa Japan; ^6^ Graduate School of Engineering and Science University of the Ryukyus Nishihara Okinawa Japan; ^7^ Suma Aqualife Park Kobe Hyogo Japan; ^8^ Tropical Biosphere Research Center University of the Ryukyus Nishihara Okinawa Japan

**Keywords:** demographic history, genomic isolation, Hydrophiini, repeat‐mask

## Abstract

Understanding the mechanisms of speciation in the open ocean, where few obvious geographic barriers exist, is an important question in ecology. Marine amniotes are an ideal model for studying speciation in the ocean because monophyly is ensured in many groups with evident time points when they have begun their life in the sea. Among such marine amniotes, sea snakes of the genus *Hydrophis* exhibit an extremely high species diversity. Here, we performed genome‐wide analyses of 16 *Hydrophis* individuals belonging to 14 species to understand how *Hydrophis* sea snakes have diversified and speciated. Demographic data suggest that most *Hydrophis* sea snakes speciated almost simultaneously around 1 million years ago, and little inter‐species admixture after speciation is inferred except for a group of genetically closely related species. Strict reproductive isolation is observed even between geographically distant conspecific local populations. The high species diversity of *Hydrophis* sea snakes may be owing mainly to such recent rapid speciation events and strict reproductive isolation.

## Graphical abstract

Understanding the mechanisms of speciation in the open ocean, where few obvious geographic barriers exist, is an important question in ecology. Here, we performed genome‐wide analyses of 16 *Hydrophis* individuals belonging to different species and found that most of the *Hydrophis* sea snakes have speciated around 1 million years ago nearly simultaneously. The high diversification rate of *Hydrophis* sea snakes may be owing mainly to such recent rapid speciation events.

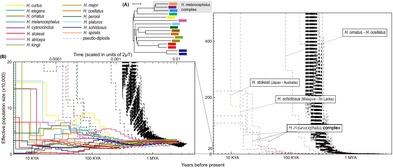

## 1 Introduction

There have been many studies about speciation in the open ocean (e.g., Kishida 2017; Kishida et al. 2021; Miya and Nishida 1997; Sanders, Lee, et al. 2013; Sanders, Rasmussen, et al. 2013) such as genetic isolation due to past climate changes (Suzuki et al. 2023) or that caused by cultural differentiation (Foote et al. 2016). However, it remains largely enigmatic how populations become separated and genetically isolated in such an open environment. Marine amniotes are an ideal model for studying speciation in the ocean because monophyly is ensured in many groups with relatively evident time points when they have begun their life in the sea. Interestingly, the species diversity differs drastically between different marine amniote groups. For example, approx. 90 extant species are described for cetaceans, whereas there are only four species described for sirenians (Jefferson et al. 2015), though both groups are considered to have shifted from land to water almost simultaneously during the Eocene (Uhen 2007).

Marine elapids (Reptilia, Squamata), collectively called sea snakes, consist of two monophyletic groups, Laticaudini and Hydrophiini. Laticaudins are oviparous and lay eggs on land, whereas hydrophiins are viviparous and spend all their life in water. They are distributed almost sympatrically in the western tropical/subtropical Pacific and are considered to have migrated from land to water independently of each other approx. 15–20 million years ago (MYA) (Lee et al. 2016), but species diversities are very different. There are only 8 species in a genus *Laticauda* described for laticaudins, whereas > 60 species in multiple genera are described for hydrophiins, though numbers of species and genera of hydrophiins remain under controversial (Cogger and Heatwole 2006; Rasmussen et al. 2011). We previously focused on laticaudins and reported that the broad distribution of major species combined with frequent inter‐species genetic admixture might have prevented laticaudins from genetic isolation and speciation (Kishida et al. 2021). In this study, we focus on hydrophiins. The high species diversity of hydrophiins owes mainly to the genus *Hydrophis* (Lee et al. 2016), in which 49 species are included (http://reptile‐database.org [accessed April 2024]; Uetz 2021). Thus, to understand the diversification of hydrophiins, it is important to understand the speciation patterns of *Hydrophis* sea snakes. There have been inconsistencies in the divergence time of the genus *Hydrophis*. Lee et al. (2016) suggested that most of the *Hydrophis* species have been speciated around 7–5 MYA. On the other hand, Sanders, Lee, et al. (2013) suggest that most of the *Hydrophis* species have become speciated recently (< 1.5 MYA). However, these studies are based only on limited genomic loci. Recently, based on the genome‐wide analyses, Ludington and Sanders (2021) inferred that the past effective population size of *Hydrophis melanocephalus* differs from that of *H. curtus* even around 10 MYA, suggesting that these two species have become reproductively structured before 10 MYA. Here, we performed genome‐wide analyses of 16 *Hydrophis* individuals belonging to 14 species in order to understand how and when *Hydrophis* sea snakes have become reproductively structured and speciated.

## 2 Materials and Methods

### 2.1 Samples

We analyzed 16 individuals belonging to 14 species of the genus *Hydrophis*, as listed in Table 1. We performed whole‐genome shotgun (WGS) sequencing of *H. ornatus* and *H. stokesii* individuals sampled in Okinawa Is., Japan (see Section 2.2). Details of the *H. stokesii* individual (specimen voucher: KUZ R88360) are described by Sasai et al. (2021). The *H. ornatus* individual (specimen voucher: KUZ R72684) was sampled in 2016 under the approval of the Suma Aqualife Park to T.S. (approval no. 0715ZA0009). In addition, we retrieved WGS reads of *Hydrophis* individuals available in the GenBank SRA database with which detailed individual information including locality is provided (14 individuals in total. SRA accession numbers are provided in Table 1). To understand not only inter‐species divergences but also intra‐species diversifications, we analyzed two conspecific individuals from different localities for *H. stokesii* and *H. schistosus* .

**TABLE 1:** Specimens analyzed in this study.

Species	Specimen voucher^a^	GenBank BioSample	SRA accession	Locality	Sex	Depth	No. of called sites [bp]	Heterozygous sites [bp]	Heterozygosity
*H. curtus*	N/A	SAMN13675263	SRR10861673	South China Sea	Female^c^	31.3	538,569,068	980,917	0.001821339
*H. elegans*	N/A	SAMN35787919	SRR24957355	Western Australia	Male^c^	74.8	585,587,290	856,186	0.001462098
*H. ornatus*	KUZ R72684	SAMD00258713^b^	DRR255413^b^	Okinawa Is., Japan	Male	18.9	543,339,950	355,153	0.000653648
*H. melanocephalus*	KUZ R72403	SAMD00134211	DRR147394	Okinawa Is., Japan	Male	81.3	585,539,300	725,281	0.001238655
*H. cyanocinctus*	N/A	SAMN12828154	SRR10617472	South China Sea	Male^c^	92.7	579,837,364	820,510	0.001415069
*H. stokesii*	KUZ R88360	SAMD00746873^b^	DRR530207^b^	Okinawa Is., Japan	Female	20.1	539,548,082	55,782	0.000103387
*H. stokesii*	N/A	SAMN42208579	SRR29672475	Exmouth, Australia	Male^c^	19.8	550,759,750	466,195	0.000846458
*H. atriceps*	N/A	SAMN42208566	SRR29672489	Gulf of Thailand	Male^c^	21.8	578,891,733	1,176,941	0.002033093
*H. kingii*	N/A	SAMN42208588	SRR29672465	Broome, Australia	Female^c^	70.7	572,189,301	549,314	0.000960021
*H. major*	N/A	SAMN42208575	SRR29672479	Broome, Australia	Male^c^	19.9	561,626,452	719,192	0.001280552
*H. ocellatus*	N/A	SAMN42208580	SRR29672474	Broome, Australia	Male^c^	21.6	559,502,620	685,963	0.001226023
*H. peronii*	N/A	SAMN42208589	SRR29672464	Pibara, Australia	Male^c^	20.6	545,612,477	451,757	0.000827981
*H. platurus*	N/A	SAMN42208574	SRR29672480	Mannar, Sri Lanka	Male^c^	20.8	557,966,852	822,116	0.001473414
*H. schistosus*	N/A	SAMN42208591	SRR29672462	Penang, Malaysia	Female^c^	22.1	568,993,188	620,794	0.001091039
*H. schistosus*	N/A	SAMN42208573	SRR29672481	Puttlam, Sri Lanka	Female^c^	25.5	562,804,344	310,424	0.000551566
*H. spiralis*	N/A	SAMN42208590	SRR29672463	Gulf of Thailand	Male^c^	40.8	573,066,168	911,107	0.001589881

^a^ KUZ: Zoological collection of the Kyoto University Museum.

^b^ Newly deposited in this study.

^c^ Judged from the autosome to Z chromosome coverage ratio.

### 2.2 DNA Extraction and Sequencing of *H. ornatus* (KUZ R72684) and *H. stokesii* (KUZ R88360)

DNA was extracted from muscle tissue of each specimen using the DNeasy Blood & Tissue Kit (QIAGEN). Paired‐end libraries were prepared using the TruSeq DNA PCR‐free Sample Prep Kit (Illumina) having an insert size of approx. 350 bp. WGS sequencing was performed on an Illumina platform with a sequencing length of 2 × 151 bp.

### 2.3 Reference Genome Sequences

A chromosome‐level genome assembly of *H. cyanocinctus* (Ludington et al. 2023) was retrieved from the GenBank (accession no. GCA_019473425.1, 1.98 Gbp). RepeatModeler (Flynn et al. 2020) v2.0.4 was employed to build a de novo repeat library of this assembly, and then RepeatMasker (http://www.repeatmasker.org) v4.1.5 was employed to mask the repetitive elements. As a result, we obtained a *H. cyanocinctus* genome assembly with 57.56% of the bases masked with “*n*.” This repeat‐masked assembly was used as a reference genome. In addition, to test the effect of repeat‐masking, we prepared the following reference sequences: the *H. cyanocinctus* genome assembly without repeat‐masking, a chromosome‐level *H. major* genome assembly (GenBank accession GCA_033807585.1, 2.17 Gbp) without repeat‐masking, and the repeat‐masked *H. major* genome assembly (61.06% of the bases were masked with “*n*”).

### 2.4 Consensus Calling

Low‐quality bases and adapters included in the paired‐end sequences of each *Hydrophis* individual (Table 1) were trimmed using fastp (Chen et al. 2018) v0.23.2, and the trimmed reads were aligned to the reference genome assembly (see Section 2.3) using the BWA‐MEM algorithm in the BWA package (Li and Durbin 2009) v0.7.17. Putative PCR‐duplicates were removed using the markdup command in the SAMtools package (Danecek et al. 2021) v1.16.1. The average alignment depth of each individual is provided in Table 1. The consensus was called using the mpileup command in the BCFtools package (Danecek et al. 2021) v1.16 following the instruction of Li and Durbin (2011) with minimum and maximum depths set to one‐third and twice the average read depth, respectively. Further calculations were performed based on the consensus sequences thus obtained. The Z chromosome was excluded from further analyses.

### 2.5 Demographic Analyses

The pairwise sequential Markovian coalescent (PSMC) method (Li and Durbin 2011) was employed to infer historical changes in the effective population size (*N*_e_). The PSMC outputs were obtained with 64 atomic time intervals and 28 free interval parameters (the “‐*p*” parameter used for PSMC was set to “4 + 25 * 2 + 4 + 6”). The genome‐wide average mutation rate (*μ*) and average generation time (*g*) of laticaudins are inferred to be *μ* = 2 × 10^−9^ [per base per year] and *g* = 5 [years] (Kishida et al. 2021). In this study, we assume these values are also applicable to hydrophiins.

A pseudo‐diploid is a diploid sequence generated in silico consisting of a haploid of an individual and that of another. Because PSMC infers past *N*_e_ values based on a frequency distribution of genetic distances between haploids, it is expected that the time point where the inferred population of a pseudo‐diploid goes to nearly infinity corresponds to the divergence time of these two individuals with the assumption of no coalescences after divergence (Li and Durbin 2011; Prado‐Martinez et al. 2013). Furthermore, the genome‐wide heterozygosity of a pseudo‐diploid gives a genetic distance (uncorrected Jukes‐Cantor distance, Jukes and Cantor 1969) between these two individuals. To derive a haploid of an individual, an allele at a heterozygous site was chosen randomly. We followed Kishida (2017) for generating pseudo‐diploid sequences. A phylogenetic tree was inferred using the neighbor‐joining (NJ) methods (Saitou and Nei 1987) based on the genome‐wide Jukes–Cantor distance matrix thus obtained, with multiple substitutions corrected assuming Poisson distribution.

### 2.6 Inferring Genomic Admixture

Inter‐species genomic admixture was assessed based on the *D*‐statistics (a.k.a. ABBA‐BABA statistics) (Green et al. 2010; Reich et al. 2010). Three species belonging to the *H. melanocephalus* complex ( *H. melanocephalus* , *H. cyanocinctus* , and *H. spiralis* , see “Section 3” for detail) were tested with *H. major* used as an outgroup. A phylogenetic tree inferred in Section 2.5 was assumed. We calculated numbers of shared alleles using the ANGSD software v0.940 (Korneliussen et al. 2014). Lower (< 30) sequencing quality bases, lower (< 30) mapping quality regions, and ambiguous/heterozygous sites in the outgroup sequence were excluded from calculation (i.e., following parameters were set for calculation: ‐minQ 30, ‐minMapQ 30, ‐enhance 1).

## 3 Results

### 3.1 Genome‐Wide Heterozygosity

**FIGURE 1:**
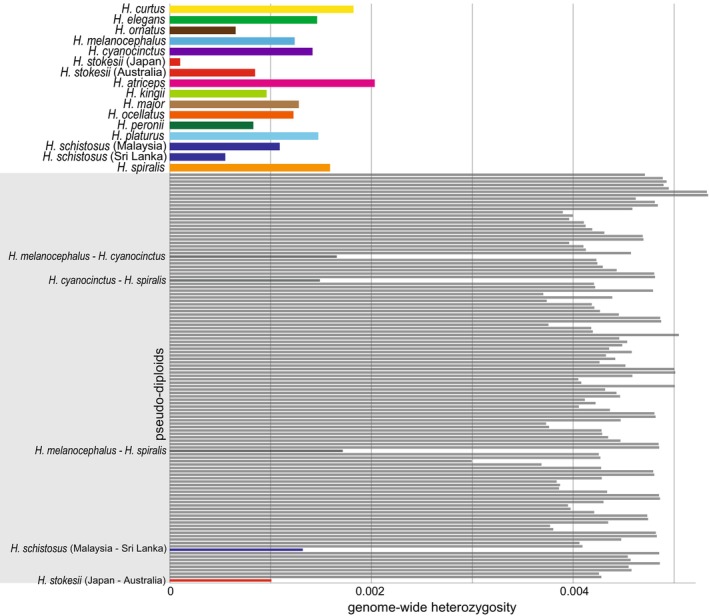
Genome‐wide heterozygosity of 16 *Hydrophis* sea snake individuals analyzed in this study (see Table 1 for detail), and that of pseudo‐diploids of all pairs of these individuals.

**FIGURE 2:**
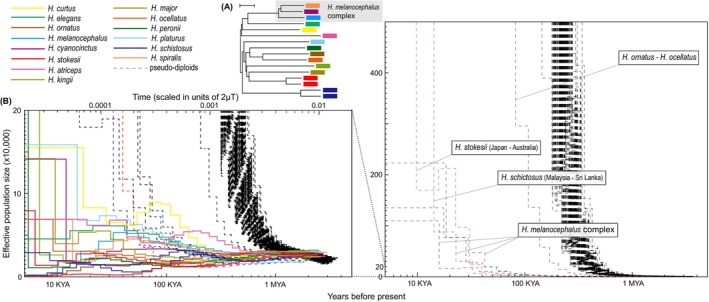
Genomic distances and demographic history of 16 *Hydrophis* sea snake individuals analyzed in this study. (A) An unrooted NJ tree of these individuals based on the genome‐wide Poisson‐corrected Jukes–Cantor distance matrix (see Table S1 for detail). Scale bar, 0.0005 substitutions/site. (B) Demographic history of *Hydrophis* sea snakes inferred with PSMC. The repeat‐masked *H. cyanocinctus* genome assembly was used as a reference genome sequence. The *x*‐axis gives time, and the lower *x*‐axis indicates scaling in years with the assumption of *μ* = 2 × 10^−9^ [per base per year]. The *y*‐axis indicates effective population size with the assumption of *μ* = 2 × 10^−9^ [per base per year] and *g* = 5 [years]. PSMC plots of the pseudo‐diploids are represented by dashed lines. *μ*, genome‐wide mutation rate [per base per year]; *T*, time before present [years].

Genome‐wide heterozygosity of 16 individuals analyzed in this study are provided in Table 1 and Figure 1. The highest is *H. atriseps* from Gulf of Thailand (0.00203), while the lowest is *H. stokesii* from Okinawa, Japan (0.000103). Genome‐wide heterozygosity of the *H. stokesii* individual from Okinawa, Japan is much lower than that of the conspecific individual from Exmouth, Australia (0.000846) despite comparable sequencing depth between them. Genome‐wide heterozygosity of pseudo‐diploids is provided in Figure 1 and Table S1. Genome‐wide heterozygosity of the pseudo‐diploids of *H. melanocephalus–H. cyanocinctus* (0.00166), *H. melanocephalus* – *H. spiralis* (0.00172), and *H. cyanocinctus–H. spiralis* (0.00149) are lower than that of an individual of some *Hydrophis* species (Figure 1). In addition, results of the *D*‐statistics suggest genetic admixture among these three species after speciation (Table 2), even if a specific phylogenetic tree ( *H. melanocephalus* , ( *H. cyanocinctus* , *H. spiralis* )) is not assumed (Table S2). We classified these genetically closely related species into a clade named “ *H. melanocephalus* complex.” Except for the conspecific pairs and pairs within the *H. melanocephalus* complex, most pseudo‐diploids show a genome‐wide heterozygosity of around 0.004–0.005 with estimated divergence times of approx. 1 MYA (Figure 1, Table S1). An NJ tree of 16 *Hydrophis* individuals based on the distance matrix calculated from the pseudo‐diploid genome‐wide heterozygosity of each pair is provided in Figure 2A.

**TABLE 2:** Sharing of derived alleles among the *H. melanocephalus* complex based on the *D*‐statistics.

H_1_	H_2_	H_3_	*n*_ABBA_	*n*_BABA_	*D* ^a^	SE^b^	*Z*‐score^c^
*H. cyanocinctus*	*H. spiralis*	*H. melanocephalus*	183,151	166,251	0.048368	0.003149	15.36

^a^ Calculated as (*n*_ABBA_ − *n*_BABA_)/(*n*_ABBA_ + *n*_BABA_).

^b^ Estimated blocked Jackknife standard error.

^c^ An absolute value of the *Z*‐score above 3 is considered as a critical value (Reich et al. 2010).

### 3.2 Demographic History of the *Hydrophis* Species

Historical changes of *N*_e_ of 16 *Hydrophis* individuals inferred with PSMC are provided in Figure 2B. Effective population sizes of all 16 individuals are estimated to have been almost equivalent before approx. 1 MYA (Figure 2). *N*_e_ plots of the pseudo‐diploids of all pairs except for those of conspecific pairs and pairs within the *H. melanocephalus* complex show similar trajectories to each other, going to excessively high values almost simultaneously. *N*_e_ plots of the intra *H. melanocephalus* complex pseudo‐diploids are inferred to have become higher than those of single *H. melanocephalus* complex individuals at around 0.05–0.1 MYA, but do not go to excessively high values. *N*_e_ plots of the conspecific pseudo‐diploids become higher values than those of conspecific single individuals around the time points where the *N*_e_ plots of two individuals become different trajectories from each other, and eventually go to excessively high values (Figure 2).

### 3.3 PSMC Analyses With Unmasked Reference Genome Assemblies

**FIGURE 3:**
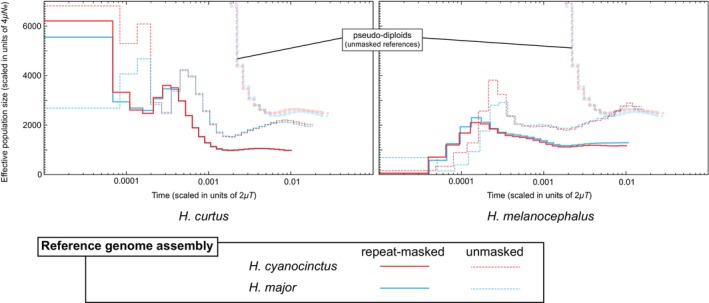
PSMC analyses of *H. curtus* (left) and *H. melanocephalus* (right) using genome assemblies of two *Hydrophis* species with/without repeat‐masking as reference genome sequences. *H. curtus–H. melanocephalus* pseudo‐diploids using unmasked *H. cyanocinctus* (red) and *H. major* (blue) reference genomes are also shown. *μ*, genome‐wide mutation rate [per base per year]; *N*_e_, effective population size; *T*, time before present [years].

In addition to the repeat‐masked *H. cyanocinctus* genome assembly, we tested the unmasked *H. cyanocinctus* genome assembly and the repeat‐masked/unmasked *H. major* genome assemblies as reference genome sequences and inferred the demographic history of *H. curtus* and *H. melanocephalus* with PSMC (Figure 3). *N*_e_ plots using unmasked reference genome assemblies differ greatly from those using repeat‐masked references. *N*_e_ plots using the repeat‐masked *H. cyanocinctus* genome assembly as a reference and those using the repeat‐masked *H. major* genome assembly show almost identical trajectories to each other. *N*_e_ plots using the unmasked *H. cyanocinctus* genome assembly and those using the unmasked *H. major* genome assembly also show similar trajectories to each other except for the recent era. *N*_e_ plots of pseudo‐diploids using unmasked references go to a very high value at around 2*μT* = 0.002 (*T* = 0.5 MYA with the assumption of *μ* = 2 × 10^−9^ [per base per year]). The number of called sites for each reference genome is provided in Table S3.

## 4 Discussion

### 4.1 Recent Nearly Simultaneous Speciation of *Hydrophis* Sea Snakes

In this study, we showed that the effective population sizes of all 16 individuals are estimated to have been equivalent before approx. 1 MYA. Because the historical *N*_e_ of the two species should be equal before the time point when the two species became genetically isolated, it is speculated that most of the *Hydrophis* species have become genetically isolated from each other around 1 MYA. *N*_e_ plots of the pseudo‐diploids also suggest that most of the *Hydrophis* species have become genetically isolated from each other at approx. 1 MYA. This estimated divergence time is much younger than those estimated in previous studies (> 5 MYA, Lee et al. 2016; Ludington and Sanders 2021), even if a lower mutation rate (1.6 MYA with the assumption of *μ* = 1.25 × 10^−9^ [per base per year]; Ludington and Sanders 2021) is assumed. Furthermore, most pseudo‐diploids except for the conspecific pairs and within the *H. melanocephalus* complex exhibit similar PSMC trajectories to each other and similar genome‐wide heterozygosity (Figures 1 and 2), suggesting nearly simultaneous speciation of most *Hydrophis* species. These pieces of evidence suggest that all *Hydrophis* species analyzed in this study, except for the *H. melanocephalus* complex, have genetically isolated and speciated nearly simultaneously around 1 MYA. Ludington et al. (2023) also speculated near‐simultaneous speciation at the root of *Hydrophis* based on the genome‐wide analyses of six individuals (five species) of *Hydrophis* sea snakes. PSMC trajectories of all pseudo‐diploids except for the *H. melanocephalus* complex go to excessively high values, suggesting strict reproductive isolation after speciation, unlike in the case of laticaudins (Kishida et al. 2021). Lee et al. (2016) and Sanders, Lee, et al. 2013 inferred not only the divergence time but the phylogenetic relationship of *Hydrophis* species based on limited genomic loci, and there are also significant inconsistencies in the phylogenetic relationships. Lee et al. (2016) inferred the following phylogenetic relationship: (((*H. ortanus*, *H. stokesii* ), ( *H. cyanocinctus* , *H. melanocephalus* )), ( *H. elegans* , *H. curtus* )). On the other hand, Sanders, Lee, et al. (2013) inferred the following tree: ( *H. curtus* , ( *H. cyanocinctus* , ( *H. ornatus* , ( *H. elegans* , *H. stokesii* )))) (Sanders, Lee, et al. (2013) did not analyze *H. melanocephalus* ). This inconsistency can be explained by the incomplete lineage sorting resulting from simultaneous speciation. Interestingly, not only hydrophiins but also sibling species of large whales distributed in the Northern and Southern Hemisphere are also speculated to have speciated at this timing (Kishida 2017). Were there any significant environmental changes that occurred at ~1 MYA in the ocean? Brunhes–Matsuyama magnetic polarity reversal occurred around this timing (approx. 0.78 MYA, Singer et al. 2019), but magnetoreception among sea snakes remains controversial (Crowe‐Riddell et al. 2019) and it is difficult to speculate why *Hydrophis* species were diversified at this time point.

### 4.2 Intra‐Species Genetic Diversity

Regarding two species ( *H. stokesii* and *H. schistosus* ), we analyzed two conspecific individuals from different localities. In both species, the PSMC trajectories of the conspecific pseudo‐diploids eventually reach excessively high values, suggesting little current gene flow between these local populations. Contrasting with laticaudins, such strict reproductive isolation between geographically distant conspecific local populations might have promoted rapid speciation of *Hydrophis* species. The genome‐wide heterozygosity of an *H. stokesii* individual sampled in Japan is significantly lower than that of other *Hydrophis* individuals, including a conspecific from Australia (Figure 1). As noted by Sasai et al. (2021), the distribution of this species had not been documented in Japan before the discovery of this individual. Although it remains unclear whether there is a novel *H. stokesii* population recently established around Japan, its reduced genetic diversity suggests that it is derived from a population with a very small effective population size, such as one on the brink of extinction or a frontier population established by a few founders.

### 4.3 The *H. melanocephalus* Complex

PSMC trajectories of the pseudo‐diploids within the *H. melanocephalus* complex suggest that these three species have become genetically isolated from each other within 0.1 MYA (Figure 2). Previous studies also suggest a close kinship between *H. melanocephalus* and *H. cyanocinctus* (Lee et al. 2016; Sanders, Rasmussen, et al. 2013). Sanders, Rasmussen, et al. (2013) suggested that *H. melanocephalus* and *H. cyanocinctus* are not monophyletic to each other based on a mitochondrial phylogenetic tree, implying immature speciation between them. Results of the *D*‐statistics suggest that there has been inter‐species admixture among them after speciation. PSMC trajectories of the intra *H. melanocephalus* complex pseudo‐diploids also suggest gene flow among them, as these trajectories do not go to excessively high values. The genome‐wide heterozygosity of the *H. melanocephalus* complex pseudo‐diploids is less than that of an individual of some *Hydrophis* species (Figure 1), indicating that inter‐specific genetic distances within the *H. melanocephalus* complex are less than intra‐specific genetic diversity of some *Hydrophis* species. All these lines of evidence imply incomplete speciation among the *H. melanocephalus* complex, raising the question of whether each of these three species should be given full species status.

### 4.4 Importance of Repeat‐Masking Upon Demographic Analyses

Notably, though Ludington and Sanders (2021) also inferred historical change of *N*_e_ of *H. melanocephalus* and *H. curtus* , their PSMC trajectories differ from those inferred in this study. The PSMC trajectories estimated using the unmasked genome assembly as a reference genome sequence differ greatly from those using the repeat‐masked genome assemblies (Figure 3), and Ludington and Sanders (2021) used unmasked genome assemblies as reference genome sequences. Inclusion of repeat regions is reported to affect the demographic inference because repeat regions are prone to a high risk of assembly errors (Patil and Vijay 2021) and should be masked from the reference sequence (Foote et al. 2016). PSMC trajectories of these two species inferred by Ludington and Sanders (2021) do not match even in an older time period (~10 MYA), suggesting that these two species have become reproductively isolated from each other to some extent even around 10 MYA. However, the PSMC trajectory of the pseudo‐diploid suggests that these two species have become genetically isolated after 2*μT* = 0.004 (*T* = 1 MYA when *μ* = 2 × 10^−9^ [per base per year] or *T* = 1.6 MYA when *μ* = 1.25 × 10^−9^ [per base per year]) even using unmasked assemblies as references (Figure 3). This inconsistency may be due to assembly and/or alignment errors in repeat regions. Although repeat regions may contain additional demographic information, the effect of the inclusion of repeat regions in the PSMC calculation is not the same between different species (Patil and Vijay 2021). Thus, to make an inter‐species comparison, we conclude that repeat regions should be excluded from the analyses to eliminate such species‐specific biases.

## 5 Conclusions

In this study, we showed that most *Hydrophis* sea snakes have speciated almost simultaneously with strict reproductive isolation around 1 MYA. Strict reproductive isolation is observed even between geographically distant conspecific local populations. This speciation pattern is in contrast to laticaudins, which have diversified gradually during 5–1 MYA with a certain amount of gene flow even after speciation (Kishida et al. 2021). The high diversification rate of hydrophiins may be owing mainly to such rapid speciation events and strict reproductive isolation.

## Author Contributions

**Takushi Kishida:** conceptualization (lead), data curation (lead), formal analysis (lead), funding acquisition (lead), investigation (lead), methodology (lead), project administration (lead), writing – original draft (lead), writing – review and editing (equal). **Rina Keboushi:** data curation (equal), writing – review and editing (supporting). **Takahide Sasai:** resources (equal), writing – original draft (supporting), writing – review and editing (supporting). **Mamoru Toda:** data curation (supporting), resources (equal), writing – original draft (supporting), writing – review and editing (equal).

## Conflicts of Interest

The authors declare no conflicts of interest.

## Supporting information


Table S1.

Table S2.

Table S3.

## Data Availability

Raw sequence reads are deposited in the SRA (BioProject PRJDB10797 [*H. ornatus*] and PRJDB17544 [*H. stokesii*]). Specimens sequenced in this study are available in the Kyoto University Museum with specimen vouchers KUZ R72684 (*H. ornatus*) and KUZ R88360 (*H. stokesii*).
